# Interaction between hypoxia, AKT and HIF-1 signaling in HNSCC and NSCLC: implications for future treatment strategies

**DOI:** 10.4155/fso.15.84

**Published:** 2016-01-29

**Authors:** Hanneke Stegeman, Paul N Span, Wenny JM Peeters, Marieke MG Verheijen, Reidar Grénman, Tineke WH Meijer, Johannes HAM Kaanders, Johan Bussink

**Affiliations:** 1 Department of Radiation Oncology, Radboud University Medical Center, P.O. Box 9101, 6500 HB Nijmegen, The Netherlands; 2 Department of Otorhinolaryngology-Head & Neck Surgery & Department of Medical Biochemistry, Turku University Hospital & University of Turku, Turku, Finland

**Keywords:** adenocarcinoma, AKT, HIF-1, HNSCC, hypoxia, NSCLC, squamous cell carcinoma

## Abstract

**Background:**

Hypoxia is a negative prognostic factor and this study investigated the relationship between hypoxia, hypoxia inducible factor 1 (HIF-1) and AKT signaling in head and neck squamous cell carcinoma (HNSCC) and non-small-cell lung cancer (NSCLC).

**Results/methodology:**

pAKT was induced by hypoxia (0.5% O_2_) in a part of HNSCC (3/4) and squamous (2/3) and adenocarcinoma (1/3) NSCLS lines. AKT-inhibitor MK-2206 reduced hypoxic HIF-1 signaling in most HNSCC cell lines. This reduction did not correlate with hypoxic induction of pAKT or with sensitivity to MK-2206 under hypoxia. Patient biopsies revealed a hypoxia-induced expression pattern of pAKT in HNSCC (n = 16), which was not observed in squamous cell (n = 34) or adenocarcinoma (n = 41) NSCLC.

**Conclusion:**

The interaction between hypoxia, HIF-1 and AKT signaling varies between tumor types and histologies, which could significantly affect response to targeted therapies.

## Abstract (lay abstract)

**Lay abstract:** Hypoxia is the result of an imbalance between oxygen delivery and oxygen consumption, and hypoxic areas are a common feature in solid tumors, including head and neck and lung cancer. Hypoxia activates various signaling pathways that enhance the survival of tumor cells (e.g., HIF-1 and AKT) and is, therefore, an inherent negative factor for outcome. In this study, we show that the interaction between hypoxia and these pathways varies widely between tumor types and histologies. This variation could significantly affect response to treatments targeting hypoxia or these signaling pathways.

**Figure 1.**
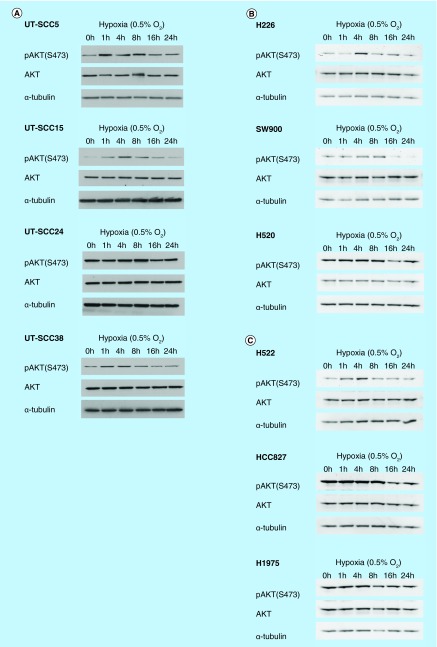
**Induction of phosphorylation of AKT by hypoxia in head and neck squamous cell carcinoma and non-small-cell lung cancer cell lines.** Expression of pAKT(S473) and AKT after incubation with hypoxia (0.5% O_2_) in **(A)** four head and neck squamous cell carcinoma; **(B)** three squamous cell carcinoma non-small-cell lung cancer and **(C)** three adenocarcinoma non-small-cell lung cancer cell lines. α-tubulin was used as loading control.

**Figure 2.**
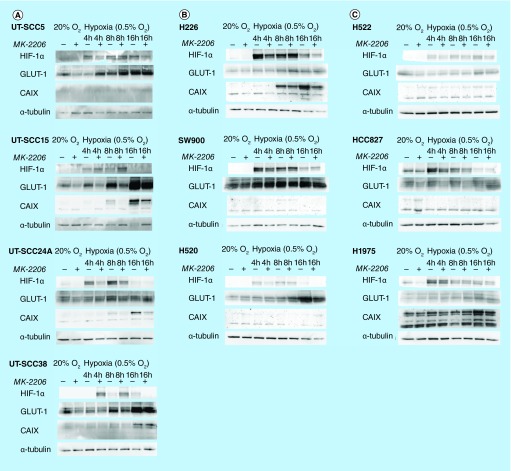
**Effect of AKT inhibition on hypoxia inducible factor 1 signaling under hypoxic conditions.** Expression of HIF-1α and downstream proteins GLUT-1 and CAIX after incubation with hypoxia (0.5% O_2_) with or without AKT inhibition (2 μM MK-2206) in **(A)** four HNSCC; **(B)** three squamous cell carcinoma NSCLC and **(C)** three adenocarcinoma NSCLC cell lines. α-tubulin was used as loading control. CAIS: Carbonic anhydrase IX; GLUT-1: Glucose transporter-1; HNSCC: Head and neck squamous cell carcinoma.

**Figure 3.**
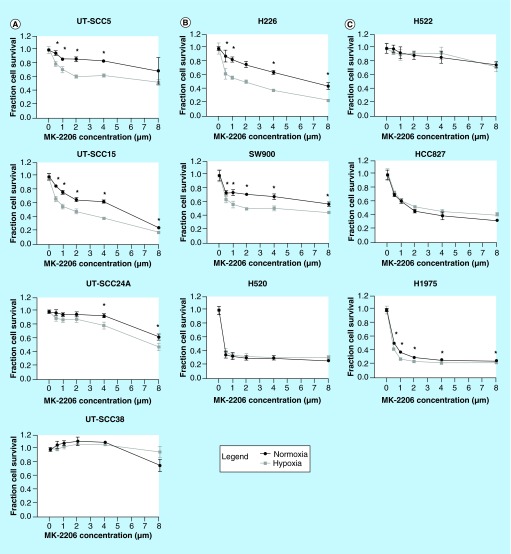
**Effect of AKT inhibition on cell survival under normoxic and hypoxic conditions.** Cell survival after treatment with MK-2206 under normoxic and hypoxic (72 h, 0.5% O_2_) conditions in **(A)** four head and neck squamous cell carcinoma; **(B)** three squamous cell carcinoma non-small-cell lung cancer and **(C)** three adenocarcinoma non-small-cell lung cancer cell lines. Cell survival was measured 72 h after hypoxic incubation in head and neck squamous cell carcinoma lines and 24 h after hypoxic incubation in non-small-cell lung cancer lines. Cell survival under hypoxic conditions shown in graph was corrected for the effect of hypoxia alone. Error bars represent standard deviation. Significant synergism between hypoxia and MK-2206 was determined by correcting the cell survival after hypoxia and MK-2206 by the effect of hypoxia alone. Differences between the effect of MK-2206 under normoxia and the corrected effect of MK-2206 under hypoxia (difference = supra-additive effect) were tested for significance using Mann–Whitney tests. Significant enhanced sensitivity for AKT inhibition under hypoxia after bonferroni correction for multiple testing is marked with an asterisk.

**Figure 4.**
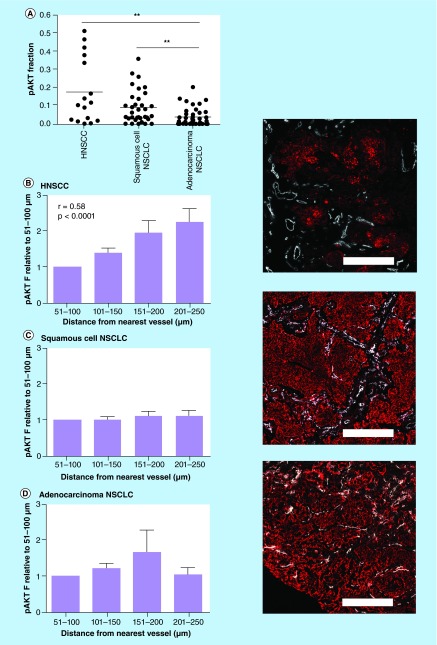
**Expression pattern of phosphorylation of AKT in patient biopsies from head and neck squamous cell carcinoma and non-small-cell lung cancer.** **(A)** Tumor fraction positive for pAKT in HNSCC and NSCLC patient biopsies. **(B)** Expression of pAKT in relation to vessels in biopsies from HNSCC; **(C)** squamous cell carcinoma NSCLC and **(D)** adenocarcinoma NSCLC patient tumors. pAKT fraction is depicted relative to the fraction observed within 51–100 μm zone from the nearest vessel. Zone within 50 μm from the vessels was excluded due to low amount of tumor cells (mostly stromal components). Microscopic images: pAKT (red), vessels (white). Magnification: 100×, scale bars represent 500 μm. HNSCC: Head and neck squamous cell carcinoma; pAKT: Phosphorylation of AKT. **p < 0.01.

Hypoxia is a common feature of solid tumors and is an inherent negative factor for treatment outcome [1]. The hypoxia-inducible factor 1 (HIF-1) pathway is an important cell survival pathway in hypoxic cells as it induces the transcription of numerous genes regulating cell proliferation, angiogenesis, glucose metabolism and apoptosis [2]. In addition to signaling via this hypoxia pathway, signaling via growth factor receptors plays an important role in hypoxic cell survival as well. Protein kinase B (AKT) is activated by hypoxia in various tumor types [3–7], and we have shown that phosphorylated AKT (pAKT) is predominantly expressed in hypoxic regions of both head and neck cancer xenograft tumors and patient biopsies [8,9]. Moreover, data of our lab indicate that hypoxic, but not normoxic, head and neck cancer cells are sensitive to AKT inhibition [9]. The interaction between hypoxia and the AKT pathway is also important for treatment response as hypoxia-induced resistance to chemotherapy has been shown to be mediated via the AKT pathway [3,6,10].

Multiple studies indicate that the interaction between the AKT pathway and HIF-1 signaling is important for hypoxic cell survival and hypoxia-induced treatment resistance [11]. The AKT pathway has been shown to enhance HIF-1 signaling by increasing HIF-1α protein levels [12–15]. Correspondingly, expression of pAKT has been correlated with the expression of HIF-1α in patient biopsies of different tumor types, including invasive breast carcinoma [16] and non-small-cell lung cancer (NSCLC) [17]. However, not all studies support this hypothesis, that is, the AKT pathway is not activated by hypoxia in all tumor cell lines and does not always mediate HIF-1 activity [18–20]. The specific interaction between hypoxia, HIF-1 and AKT signaling in a tumor could play an important role in the response to various new treatment strategies, including hypoxic modification and AKT pathway inhibitors. Hence, insight in the interaction between hypoxia and AKT signaling could help to optimize patient selection and consequently patient outcome.

In this study, we investigated the relationship between hypoxia, AKT activation and HIF-1 signaling in a head and neck squamous cell carcinoma (HNSCC) cell line panel. A panel of NSCLC lines consisting of both adenocarcinomas and squamous cell carcinomas was also studied to determine the variation between tumor types and histologies within one tumor type. Moreover, the effect of AKT inhibition on hypoxic cell survival was tested to increase the insight in the dependence of hypoxic cell survival on AKT activity. Lastly, the expression pattern of pAKT was studied in patient biopsies from HNSCC and NSCLC to assess whether the observed *in vitro* relations between AKT activation and hypoxia are also observed in the clinic.

## Material & methods

### Cell lines

Four human HNSCC cell lines (UT-SCC lines, generated by R.G., University of Turku) and six human NSCLC cell lines (obtained from the ATCC) were used in this study. The characteristics of the cell lines are shown in Table 1. Cell lines were not further authenticated or tested. Cells were cultured in T75 culture flasks, under humidified conditions (37ºC, 5% CO_2_). Cells were maintained in DMEM (HNSCC lines) or RPMI1640 (NSCLC lines) containing 2 mM l-glutamine, 1% nonessential amino acids, 20 mM Hepes, 10 units/ml penicillin, 10 units/ml streptomycin and 10% fetal bovine serum.

### Hypoxic incubation & MK-2206 treatment

To determine protein expression after hypoxia, cells were incubated under hypoxic conditions (0.5% O_2_, H35 hypoxystation, Don Whitley Scientific Ltd., West Yorkshire, UK) for 1 h, 4 h, 8 h, 16 h or 24 h. To determine protein expression after AKT inhibition, cells were treated overnight (16 h) with 0 or 2 μM MK-2206 (Selleckchem, Houston, TX, USA) under standard normoxic conditions and thereafter incubated under normoxic conditions or under hypoxic conditions (0.5% O_2_, H35 hypoxystation, Don Whitley Scientific Ltd., West Yorkshire, UK) for 4 h, 8 h or 16 h.

To assess cell survival after hypoxia and/or AKT-inhibition, cells were seeded in 96-well plates. After the cells were allowed to attach overnight under standard normoxic conditions, 0, 0.5, 1, 2, 4 or 8 μM, MK-2206 was added and cells were incubated under normoxic conditions or under hypoxic conditions (0.5% O_2_) for 72 h. To prevent interference of hypoxia in the determination of cell survival with the cell-counting Kit-8 assay (Sigma–Aldrich Chemie BV, Zwijndrecht, The Netherlands), cells were placed back into normoxic conditions before measurement of cell survival. Cell survival was measured 72 h after hypoxic incubation in HNSCC lines and 24 h after hypoxic incubation in NSCLC lines.

### Western blot analysis

Cells were lysed in radioimmunoprecipitation assay (RIPA) buffer and protein was quantitated using a standard Bradford absorbance assay. Proteins were separated by SDS-PAGE and blotted onto polyvinylidene fluoride (PVDF) membrane. Membranes were incubated with the appropriate primary antibodies followed by incubation with horseradish peroxidase (HRP)-conjugated antibodies. Finally, proteins were detected using chemiluminescence. Antibodies against the following antigens were used: HIF1-alpha (BD-Transduction Laboratories, San Jose, CA, USA), GLUT-1 (H-43; Thermo Scientific, Waltham, MA, USA), CAIX (D47G3; Cell Signaling Technology), pAKT(S473; Cell Signaling Technology), AKT 1/2/3 (H-136; Santa Cruz Biotechnology, Santa Cruz, CA, USA), HRP-conjugated goat-antirabbit IgG (Cell Signaling Technology), HRP-conjugated goat-antimouse IgG (Santa Cruz Biotechnology, Santa Cruz, CA, USA) and α-tubulin (Calbiochem, San Diego, CA, USA). Given the many intricacies in quantifying Western Blots, we chose only to make qualitative statements on the intensities of the stainings.

### Patient biopsies

Biopsies were obtained from 16 patients with stage I–IV squamous cell carcinoma of the oral cavity or oropharynx planned for curative treatment with fractionated radiotherapy with or without concurrent chemotherapy. Before onset of treatment, biopsies were taken for routine diagnostic purposes and additional biopsies were taken for multiple marker analyses. The latter were snap-frozen in liquid nitrogen and used in this study.

Fresh frozen biopsies of 75 NSCLC tumors of a total of 74 patients were included from a study described in detail by Meijer *et al*. [21]. Only tumors with the histology of adenocarcinoma and squamous cell carcinoma were used for this study.

All patient studies were performed according to institutional ethical rules and regulations.

### Immunohistochemical staining, image acquisition & analysis of tumor sections

To determine localization of protein expression *in vivo*, frozen tumor sections (5 µm) were thawed, fixed in acetone (4ºC) and rehydrated in phosphate-buffered saline (PBS). Tumor sections were stained for pAKT in combination with vessels. The antibody against pAKT(S473) was obtained from Santa Cruz Biotechnology and the antihuman endothelium antibody PAL-E was obtained from Euro Diagnostica AB (Nijmegen, The Netherlands). pAKT was detected by incubation with Cy3-conjugated goat-antirabbit F(ab’)_2_ fragments (Jackson Immuno Research Laboratories Inc., West Grove, PA, USA), and PAL-E by incubation with Alexa647-conjugated chicken-antimouse IgG (Molecular Probes, Leiden, The Netherlands). Stained sections were mounted in Fluorostab (ICN Pharmaceuticals, Inc, Zoetermeer, The Netherlands).

Stained tumor sections were scanned on a digital image processing system on a fluorescence microscope (Axioskop, Zeiss, Göttingen, Germany) and a computer-controlled motorized stepping stage, using IPLab software. Each section was sequentially scanned at 100× magnification, yielding an image of pAKT-expression and an image of the vasculature (PAL-E).

One pseudocolored composite image was reconstructed from the individual microscope images. Using this composite image and a hematoxylin-eosin stained tumor section, total tumor area was delineated and nontumor tissue, necrotic area and staining artifacts were excluded from the analysis. Thereafter, thresholds for the fluorescence signals were interactively set at intensities where the steepest gradient occurred between background and foreground intensity levels, and gray value images were converted to binary images. To quantify the distribution of pAKT in relation to the vasculature, zones were chosen at increasing distances from the nearest vessel (0–50, 51–100, 101–150, 151–200 and 201–250 μm). Using Image J software (NIH, Bethesda, MD, USA), the fraction positive for pAKT in each zone was calculated by dividing the zone area positive for pAKT by the total zone area. Zones with areas <200 pixels were excluded from the analysis.

### Statistics

Statistical analyses were performed using Prism (GraphPad Software, Inc., LA Jolla, CA, USA) or SPSS (SPSS, Chicago, IL, USA). p-values < 0.05 were considered significant. Synergism between hypoxia and MK-2206 was determined by correcting the cell survival after hypoxia and MK-2206 by the effect of hypoxia alone. Differences between the effect of MK-2206 under normoxia and the corrected effect of MK-2206 under hypoxia (difference = supra-additive effect) were tested for significance using Mann–Whitney tests. Bonferroni correction was applied to correct for multiple testing. Correlations between parameters were assessed using the Spearman correlation test.

## Results

### pAKT is induced by hypoxia in HNSCC cells & to a lesser extent in NSCLC cells

We have shown before that hypoxia increases pAKT expression in two HNSCC cell lines [9]. To obtain insight into whether this is a more common phenomenon in HNSCC, we extended our cell panel to four HNSCC lines and determined the effect of hypoxia (0.5% O_2_) on pAKT levels in a time-dependent manner (Figure 1A). In addition, six NSCLC lines were studied to explore the variation between tumor types and histologies; three squamous cell carcinoma lines (Figure 1B) and three adenocarcinoma lines (Figure 1C) of the lung were investigated. Characteristics of the cell lines are shown in Table 1.

As observed before, pAKT expression increased by incubation with 0.5% O_2_ in a time-dependent manner in all HNSCC lines, except for UT-SCC24A. In UT-SCC5, -15 and -38, pAKT levels increased within 1 h of hypoxic incubation, and decreased to levels observed in normoxic cells after 16 h. Although less evident, two out of three (H226 and SW900) squamous cell lines and one out of three (H522) adenocarcinoma lines of the lung showed upregulation of pAKT under hypoxia. Interestingly, both the HNSCC and the NSCLC cell lines that did not upregulate pAKT had already high levels of pAKT under normoxic conditions. In almost all cell lines a decrease of pAKT at later incubation times was seen irrespective of the induction of AKT by hypoxia. Although clear differences between cell lines of the same tumor type and histology were observed, these data suggest that the AKT pathway is activated by hypoxia in HNSCC and to a lesser extent in NSCLC.

### Interaction between AKT activity & hypoxic HIF-1 signaling is not correlated with induction of pAKT by hypoxia

To determine whether HIF-1 signaling under hypoxic conditions is regulated by the AKT pathway in the tested cell lines, the expression levels of HIF-1α and downstream proteins glucose transporter-1 (GLUT-1) and carbonic anhydrase IX (CAIX) were investigated under hypoxia with or without addition of the allosteric AKT inhibitor MK-2206 (Figure 2). Increased expression of HIF-1α under hypoxia was observed in all cell lines, which most often was followed by increased levels of GLUT-1 and CAIX (Figure 2). GLUT-1 and CAIX were not induced in all cell lines; for example, UT-SCC24A did not show upregulation of GLUT-1, while HCC827 hardly showed any upregulation of CAIX. pAKT levels were decreased by MK-2206 during normoxic and hypoxic incubation in all cell lines indicating effective AKT inhibition (Supplementary Figure 1).

AKT inhibition decreased the hypoxic induction of GLUT-1 and/or CAIX in HNSCC lines UT-SCC15, -24A and -38. Whereas in UT-SCC24A this was associated with a concomitant decrease in HIF-1α, in UT-SCC15 and -38 HIF-1α levels increased after AKT inhibition. In UT-SCC5 reduced hypoxic HIF-1α levels were observed after AKT inhibition, but this did not result in reduced GLUT-1 or CAIX levels. Overall, no unequivocal effects of AKT inhibition on HIF-1 signaling were found in these lines. Interaction of the AKT and HIF-1 pathway under hypoxia was not correlated with hypoxic induction of pAKT in these HNSCC cell lines (Figure 1A). In general, no effects of AKT inhibition on HIF-1 signaling were observed in either squamous cell carcinoma or adenocarcinoma NSCLC lines (Figure 2B & C). AKT inhibition only reduced HIF-1 and CAIX levels at 16 h of hypoxia in H226.

Overall, these data show that the AKT pathway regulates hypoxia-induced HIF-1 signaling in part of the HNSCC lines and that this regulation is independent of hypoxic-induction of pAKT. No interaction between the AKT pathway and hypoxia-induced HIF-1 signaling was observed in the NSCLC lines.

### Hypoxic sensitization to AKT inhibition is observed in HNSCC & NSCLC lines and is independent of effects of AKT inhibition on HIF-1 signaling

We have shown before that cell survival of UT-SCC5 and UT-SCC15 was decreased by AKT inhibition under hypoxic, but not normoxic conditions [9]. Hypoxic sensitization to AKT inhibition could be due to the interaction of the AKT and HIF-1 pathway under hypoxic conditions. To test this hypothesis, we determined the sensitivity of our cell line panel to a series of MK-2206 concentrations under both normoxic and hypoxic conditions (Figure 3). To determine whether hypoxic cells showed a higher sensitivity for MK-2206, cell survival after hypoxia and MK-2206 was corrected by the effect of hypoxia alone. Significant differences between the effect of MK-2206 under normoxia and the corrected effect of MK-2206 under hypoxia indicate that hypoxic cells are more sensitive to AKT inhibition (significant differences are marked with asterisks in Figure 3).

As shown before, hypoxic cells of UT-SCC5 and UT-SCC15 were more sensitive to MK-2206 compared with normoxic cells (Figure 3A). In contrast, UT-SCC24A and UT-SCC38 showed very low sensitivity to MK-2206 under both normoxic and hypoxic conditions (Figure 3A). In UT-SCC24A, the survival of hypoxic cells was significantly higher than normoxic cells (p = 0.0022). Of note, this cannot be seen here, as cell survival is shown corrected for the effect of hypoxia alone. The enhanced survival after hypoxia of the UT-SCC 24A resulted in significant enhanced sensitivity for MK-2206 under hypoxia at the highest doses in this figure. However, in absolute sense the survival of normoxic and hypoxic cells was comparable at these doses. Squamous cell NSCLC lines H226 and SW900 also showed a higher sensitivity to MK-2206 under hypoxia compared with normoxia, whereas H520 cells showed a high sensitivity for MK-2206 independent of hypoxia (Figure 3B). Two adenocarcinoma NSCLC lines (HCC827 and H1975) showed high sensitivity and one (H522) low sensitivity for MK-2206 (Figure 3C). Although the difference was small, hypoxic H1975 cells did show enhanced sensitivity for MK-2206 compared with normoxic cells.

An overview of the data from Figures 1–Figure 3 is shown in Table 1. Enhanced sensitivity for AKT inhibition under hypoxia was observed in part of HNSCC and NSCLC lines, irrespective of the effect of AKT inhibition on hypoxic HIF-1 signaling.

### pAKT expressed in hypoxia-induced pattern in HNSCC, but not in NSCLC patient biopsies

Tumor biopsies from 16 HNSCC and 74 NSCLC patients were stained for pAKT and vessels to visualize the expression pattern of pAKT. Patient characteristics are shown in Table 2. Overall, the fraction of the tumor positive for pAKT was significantly higher in the squamous cell carcinomas of both the head and neck and lung compared with the adenocarcinomas of the lung (Figure 4A). In HNSCC biopsies, pAKT expression increased with larger distance from the vessels indicating that pAKT expression increased in areas with lower oxygen tension (Figure 4B). This resulted in a significant correlation between the relative pAKT fraction and distance from the vessels (r_s_ = 0.58; p < 0.0001). In contrast, in NSCLC biopsies, either squamous cell or adenocarcinoma, pAKT expression did not increase with increasing distance from the vessels; that is, pAKT was expressed throughout the tumor or not (Figure 4C & D). Correspondingly, no significant correlation between the relative pAKT fraction and distance from the vessels was observed in both histologies.

## Discussion

Hypoxia and growth factor receptor signaling are known factors that cause resistance to anticancer treatments in solid tumors, including chemotherapy and radiotherapy. Reduction of these factors has the potential to significantly improve treatment response and outcome. However, strategies to reduce hypoxia and growth factor receptor signaling are often only effective in a part of the patient population. There are indications that the interaction between hypoxia and growth factor receptor signaling is important for treatment response to these new strategies [22]. Increased insight in these interactions could thus improve treatment choice and consequently treatment outcome.

The involvement of the AKT pathway in the regulation of HIF-1 signaling under hypoxia is controversial as contradicting results have been reported. In this study we investigated an extensive panel of HNSCC and NSCLC lines. Hypoxic induction of pAKT was most often observed in the HNSCC lines, but also to a lesser extent in the NSCLC lines. The lack of upregulation of pAKT in NSCLC could be due to the fact that these cell lines showed more often high pAKT levels under normoxic conditions, which could not be further upregulated by hypoxia. Upregulation of pAKT was highest at 4–8 h of hypoxia and thereafter pAKT levels decreased. This downregulation of pAKT at later time points was also observed in cell lines that did not show upregulation of pAKT. Downregulation of pAKT during chronic hypoxia has also been observed by Mottet *et al*. [23] in HepG2 hepatoma cells. In that study inhibition of the AKT pathway did result in decreased HIF-1α at earlier time points, but not at later time points. In our study we did not observe this biphasic effect on HIF-1α expression. However, we did show that the effect of AKT inhibition on HIF-1 signaling is not dependent on the induction of pAKT by hypoxia. Blancher *et al*. [24] similarly showed effects of AKT pathway inhibition on HIF-1α and VEGF expression during hypoxia without induction of pAKT by hypoxia in a panel of breast cancer cell lines. Downregulation of hypoxic HIF-1 signaling by AKT inhibition was most clearly observed in two out of four HNSCC lines. Only a small reduction of HIF-1 signaling was observed in one out of six NSCLC lines, which indicates that the AKT pathway is not an important regulator of hypoxic HIF-1 signaling in NSCLC.

Enhanced sensitivity to AKT inhibition under hypoxia was observed in part of the HNSCC and NSCLC lines. Although regulation of HIF-1 signaling by AKT was observed in HNSCC, hypoxic sensitization to AKT inhibition was not correlated with the effect of AKT inhibition on hypoxic HIF-1 signaling. In contrast, hypoxic sensitization to AKT inhibition was, except for H1975, only observed in cell lines that showed upregulation of pAKT under hypoxia. This observation indicates that enhanced activity of the AKT pathway under hypoxia is important for hypoxic cell survival in these cell lines, but that this protective effect of AKT is independent on HIF-1 signaling. The AKT pathway induces multiple cell survival processes, including inhibition of apoptosis, which results in improved survival under stressful conditions [25]. In addition, the AKT pathway interacts with the mTOR and the unfolded protein response pathways that also play a major role in hypoxic cell signaling and survival [26,27]. Further research will be necessary to determine which pathways downstream of AKT are important for hypoxic sensitization to AKT inhibition. As observed in this study, the interaction between the AKT pathway and the various pathways is likely to be cell-type dependent and influenced by microenvironmental parameters like glucose concentration and the availability of growth factors. We can also not exclude that decreased HIF-1 signaling by AKT inhibition does play a role in the enhanced sensitivity for AKT inhibition under hypoxia in a part of our cell lines. Furthermore, UT-SCC38 and H522 showed upregulation of pAKT during hypoxia, but did not show enhanced sensitivity to AKT inhibition under hypoxia. This indicates that AKT-independent activation of downstream pathways could cause resistance to AKT inhibition in hypoxic cells.

Interestingly, the most of the tested HNSCC lines showed a relatively low sensitivity to AKT inhibition, while some NSCLC showed a very high sensitivity to AKT inhibition. The NSCLC lines showing high sensitivity to AKT inhibition, that is, H520, HCC827 and H1975, have also the highest basal pAKT levels. The high activity of AKT thus seems to reflect a dependence on the AKT pathway for cell survival in these cell lines. In the NSCLC lines with low basal pAKT levels that induced pAKT under hypoxia, that is, H226 and SW900, showed enhanced sensitization to AKT inhibition under hypoxia indicating that the importance of the AKT pathway for cell survival can become larger under stressful conditions. In the HNSCC lines the constitutive expression of pAKT was not correlated with sensitivity for AKT inhibition. This discrepancy between the NSCLC and HNSCC lines could be due to a differential genetic background. In general, NSCLC have more often activating mutations in important growth factor receptor signaling genes like EGFR and KRAS, although the percentage is lower in squamous cell NSCLC compared with adenocarcinoma NSCLC [28,29]. These activating mutations can result in ‘oncogene addiction’, which can make a tumor cell very sensitive to inhibition under control conditions. The AKT pathway is an important downstream pathway of both EGFR and KRAS and cell lines with mutations in these genes could thus also be dependent on AKT signaling. We have not performed mutational analysis in our cell panel, but it is for example known that H1975 and HCC827, both very sensitive for AKT inhibition, have activating mutations in EGFR [30]. Consistent with the *in vitro* results pAKT was expressed in a hypoxia-related pattern in the HNSCC biopsies. These results confirm our previous observations in larger cohorts of HNSCC patients [8,31]. Hypoxic induction of pAKT was only observed in two out of six NSCLC lines. In some NSCLC biopsies a hypoxia-related pAKT expression was visible, but as this was not a general expression pattern there was no overall correlation between the pAKT expression and distance from the vessels observed in this patient population. Hence, our *in vitro* observations do seem to reflect the clinical situation.

The observed differences in the interaction between hypoxia, AKT and HIF-1 signaling between tumors of different origin and histology could have important consequences for patient selection for AKT inhibition and hypoxic modification. Our data show that high AKT expression in NSCLC, either present due to mutational changes or induced by hypoxia, is correlated with high sensitivity to AKT inhibition. This suggests that AKT inhibition is a promising target to improve outcome in NSCLC patients with high pAKT expression. Moreover, NSCLC patients with hypoxic tumors could additionally benefit from hypoxic modification or HIF-1 inhibition, as there was no interaction between AKT and HIF-1 signaling in this tumor type. Hypoxic HNSCC cells that upregulated pAKT showed the highest sensitivity to AKT inhibition indicating that AKT inhibition could be beneficial in HNSCC patients with pAKT expressed in a hypoxia-related pattern. AKT inhibition, however, was not successful in all HNSCC lines that upregulated pAKT under hypoxia and further research will be necessary to find additional tumor characteristics that could predict for treatment response to AKT inhibition under hypoxia to identify this patient group.

## Conclusion

The interaction between hypoxia, AKT activation and HIF-1 signaling varies widely between tumor types and cell lines of the same tumor type. In the cell lines tested here, an interaction between AKT signaling and hypoxic HIF-1 signaling was observed in squamous cell carcinoma of the head and neck, but not in squamous cell or adenocarcinomas of the lung. However, this variation could not explain the differential response to AKT inhibition under normoxic and hypoxic conditions. Further research will be necessary to identify which pathways are critical for response to both targeted therapies like AKT inhibition and hypoxic modification. Only in this way predictive biomarkers can be identified and patient selection can be optimized to obtain the best clinical outcome for each individual patient.

## Future perspective

Increasing insight in mechanisms that induce resistance to anticancer treatments in solid tumors has led to the development of various therapies that specifically target these resistance mechanisms. However, clinical success is often reduced because these strategies are only effective in a part of the patient population. Hence, insight in the factors that influence treatment effectiveness could help to optimize patient selection and consequently patient outcome. This study investigated the interaction between hypoxia, HIF-1 and AKT signaling, which are tumor factors that could play an important role in the response to hypoxic modification and AKT pathway inhibitors. We show that this interaction varies widely between tumor types and cell lines of the same tumor type. These differences could affect treatment response to various anticancer strategies significantly. Our observations underwrite the high variation in critical tumor characteristics and importance for future research into predictive biomarkers. Only in this way patient selection can be optimized, which will significantly increase the chance on clinical success of newly developed anticancer therapies.

**Table 1.** **Overview of cell line characteristics and interaction between hypoxia, AKT and hypoxia inducible factor 1 signaling.**

**Cell line**	**Tumor type**	**Histology type**	**Hypoxic induction of pAKT**	**Downregulation of HIF-1 signaling by AKT inhibition**	**Enhanced sensitivity to AKT inhibition under hypoxia**
UT-SCC5	HNSCC	Squamous cell	++	-	++
UT-SCC15	HNSCC	Squamous cell	++	++	++
UT-SCC24A	HNSCC	Squamous cell	-	++	-/+
UT-SCC38	HNSCC	Squamous cell	++	-	-
H226	NSCLC	Squamous cell	+	+	++
SW900	NSCLC	Squamous cell	+	-	++
H520	NSCLC	Squamous cell	-	-	-
H522	NSCLC	Adenocarcinoma	+	-	-
HCC827	NSCLC	Adenocarcinoma	-	-	-
H1975	NSCLC	Adenocarcinoma	-	-	+

++: Strongly present; +: Present ; -/+: Marginal; -: Negative; HIF-1: Hypoxia inducible factor 1; HNSCC: Head and neck squamous cell carcinoma; NSCLC: Non-small-cell lung cancer; pAKT: Phosphorylation of AKT.

**Table 2.** **Tumor characteristics of patient biopsies.**

**Characteristic**	**Number of tumors**
**Head and neck squamous cell carcinoma tumor characteristics**	
Tumor site:	
− Oropharynx	15
− Oral cavity	1
T classification:	
− T1	0
− T2	6
− T3	5
− T4	5
N classification:	
− N0	4
− N+	12
**Non-small-cell lung cancer tumor characteristics**	
Histology:	
− Adenocarcinoma	41
− Squamous cell carcinoma	34
T classification:	
− T1	27
− T2	38
− T3	4
− T4	6
N classification:	
− N0	45
− N+	30

**Executive summary**

Phosphorylation of AKT (pAKT) is induced by hypoxia in head and neck squamous cell carcinoma (HNSCC) cells and to a lesser extent in NSCLC cells.
Interaction between AKT activity and hypoxic hypoxia inducible factor 1 (HIF-1) signaling is not correlated with induction of pAKT by hypoxia.
Hypoxic sensitization to AKT inhibition is observed in HNSCC and NSCLC lines and is independent of effects of AKT inhibition on HIF-1 signaling.
pAKT is expressed in a hypoxia-induced pattern in HNSCC, but not in NSCLC patient biopsies.
The interaction between hypoxia, AKT activation and HIF-1 signaling varies widely between tumor types and cell lines of the same tumor type, which could have important consequences for response to treatments targeting hypoxia or growth factor receptor signaling.
