# Supplementary figures and images for: Interaction between hypoxia, AKT and HIF-1 signaling in HNSCC and NSCLC: implications for future treatment strategies
Source: Future Sci OA. 2016 Jan 29;2(1):FSO84. doi: 10.4155/fso.15.84 (PMC5137923; doi:10.4155/fso.15.84)

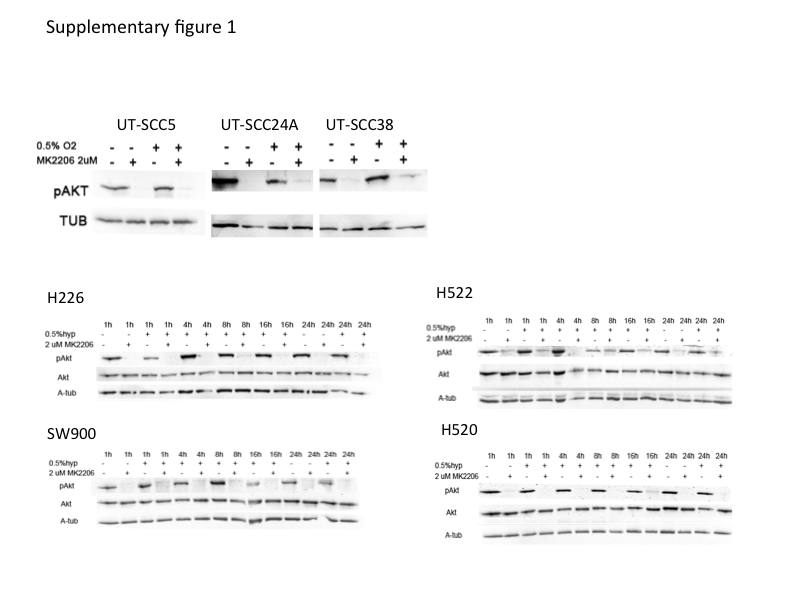

Supplement: Supplementary file 1 [file fso-02-84-s1.tif]
